# The association between intraoperative urine output and postoperative acute kidney injury differs between partial and radical nephrectomy

**DOI:** 10.1038/s41598-018-37432-7

**Published:** 2019-01-24

**Authors:** Min Hur, Sun-Kyung Park, Seokha Yoo, Sheung-Nyoung Choi, Chang Wook Jeong, Won Ho Kim, Jin-Tae Kim, Cheol Kwak, Jae-Hyon Bahk

**Affiliations:** 1 Department of Anesthesiology and Pain Medicine, Seoul National University Hospital, Seoul National University College of Medicine, Seoul, South Korea; 2 Department of Urology, Seoul National University Hospital, Seoul National University College of Medicine, Seoul, South Korea

## Abstract

We sought to investigate the association between intraoperative urine output and postoperative acute kidney injury (AKI) in patients undergoing radical and partial nephrectomy. We retrospectively reviewed data of 742 patients. Postoperative AKI was defined by the Kidney Disease: Improving Global Outcomes criteria. The relationship between intraoperative urine output and the risk of AKI was evaluated by multivariable logistic regression analysis in radical and partial nephrectomy, separately. Minimum *P*-value approach was used to find the optimal threshold of intraoperative oliguria associated with the risk of AKI. The incidence of AKI was 14.4% (67/466) after partial nephrectomy and 57.6% (159/276) after radical nephrectomy. For partial nephrectomy, multivariable analysis showed that renal ischemic time, operation time, open surgery and intraoperative transfusion were significantly associated with AKI. For radical nephrectomy, history of hypertension, baseline glomerular filtration rate and intraoperative mean urine output were significantly associated with AKI. Intraoperative mean urine output during radical nephrectomy was associated with AKI after radical nephrectomy, while not after partial nephrectomy. Mean urine output <1.0 mL/kg/h was determined to be an optimal cutoff of AKI after radical nephrectomy. Intraoperative oliguria may have different clinical implication for AKI between partial and radical nephrectomy.

## Introduction

The incidence of acute kidney injury (AKI) after partial nephrectomy has been reported to be still as high as 54%^1,2^, although the AKI after nephrectomy is considerably different from the general postoperative AKI because estimated glomerular filtration rate (eGFR) reduction after nephrectomy include both the renal mass reduction and damage on the remnant kidney^3,4^. Nephrectomy-induced chronic renal insufficiency is associated with increased postoperative mortality^5^ and new baseline GFR can impact survival after nephrectomy^4,6^. As postoperative AKI is associated with the development of chronic kidney disease^7–9^, the AKI after nephrectomy may be associated with poor patient survival. However, the potential impact of AKI after nephrectomy on patient outcomes has not been clearly defined. Only a few studies investigated the impact of AKI on long-term renal outcomes^10^ and perioperative factors that are associated with renal dysfunction after nephrectomy^1,4^.

Both the short-term and long-term postoperative renal function significantly decreases after nephrectomy^1,2,11^, although most kidneys eventually recover their function and immediate decline in eGFR after nephrectomy does not impact on patient prognosis^2,4^. Regarding long-term effect, Krebs *et al*.^11^ evaluated the decreased renal function in 12 months after nephrectomy and reported that renal function significantly decrease and 4.6% of patients progressed to end-stage renal disease after radical nephrectomy. Reported risk factors associated with progressive chronic kidney disease after nephrectomy include radical nephrectomy, patient age, preoperative proteinuria, and baseline eGFR^11–14^. For short-term effect, Rajan *et al*. reported that 39% of patients developed AKI after partial nephrectomy during four days after surgery^1^. Zhang *et al*. reported 46% as the incidence of AKI after partial nephrectomy according to their proposed criteria during the immediate postoperative period^2^. AKI was significantly associated with functional recovery during 4 to 12 months after surgery. Therefore, diagnosis, prevention, and management of AKI after partial or radical nephrectomy might be important to maintain residual renal function after nephrectomy.

AKI is diagnosed by clinical criteria including RIFLE, AKIN, and KDIGO criteria^15^. All criteria involve a serum creatinine elevation after surgery and oliguria with a cutoff of 0.5 or 0.3 mL/kg/hr. Intraoperative urine output is influenced by many factors including hemodynamics, sympathetic tone, intra-abdominal pressure, aldosterone and antidiuretic hormone level. Indeed, previous studies reported a different cutoff of oliguria that is associated with acute kidney injury after surgeries other than nephrectomy^16–18^. A conventional cutoff of defining oliguria (<0.5 or 0.3 ml/kg/h) appears to be less reliable to predict acute kidney injury (AKI) in the surgical settings. The fluid management strategy during nephrectomy has not been well characterized or evaluated in the previous studies, and intraoperative fluid administration during nephrectomy is still performed under the guidance of urine output. Therefore, it is important to investigate the association between intraoperative urine output and the risk of AKI after partial or radical nephrectomy. However, the association between oliguria and AKI as well as an optimal cutoff of oliguria might be different between partial and radical nephrectomy due to the different surgical time, bleeding amount, or intraoperative mannitol infusion.

The purpose of this retrospective study was to investigate the relationship between perioperative variables including intraoperative urine output and the risk of postoperative AKI in patients undergoing radical and partial nephrectomy. We attempted to find the optimal cutoff of oliguria that is associated with the risk of AKI after radical and partial nephrectomy. We performed the analysis for radical and partial nephrectomy separately due to different surgical and anesthetic conditions and possible differences in the distribution of urine output between radical and partial nephrectomy.

## Results

Patient characteristics and perioperative variables were compared in Table 1. More patients received laparoscopic surgery for radical nephrectomy, while more patients received robot-assisted surgery for partial nephrectomy. The patients who underwent radical nephrectomy had poorer baseline renal function and frequent proteinuria.

**Table 1:** Patient characteristics and perioperative parameters.

Characteristic	Partial nephrectomy	Radical nephrectomy	P-value
Patient population, n	466	276	
Demographic data			
Age, years	56 (47–65)	61 (52–69)	<0.001
Female, n	128 (27.5)	80 (29.0)	0.656
Body-mass index, kg/m^2^	24.5 (22.5–26.7)	24.4 (22.5–26.3)	0.169
Background medical status			
Hypertension, n	175 (37.6)	139 (50.4)	0.001
Diabetes mellitus, n	65 (13.9)	48 (17.4)	0.207
Cerebrovascular accident, n	11 (2.4)	4 (1.4)	0.394
Preoperative hemoglobin, g/dl	14.1 (12.9–15.1)	13.4 (12.0–14.6)	<0.001
Preoperative serum albumin level, mg/dl	4.4 (4.2–4.6)	4.3 (4.0–4.5)	<0.001
Preoperative serum creatinine, mg/dL	0.90 (0.79–1.04)	0.96 (0.82–1.12)	<0.001
Preoperative GFR, calculated by MDRD	83 (72–95)	76 (64–87)	<0.001
Preoperative stage of CKD			
GFR ≥ 90 mL/min/1.73 m^2^	158 (33.9)	57 (20.7)	<0.001
60 ≤ GFR < 89 mL/min/1.73 m^2^	276 (59.2)	163 (59.1)	
45 ≤ GFR < 59 ml/min/1.73 m^2^	25 (5.4)	41 (14.9)	
30 ≤ GFR < 44 ml/min/1.73 m^2^	7 (1.5)	15 (5.4)	
Preoperative proteinuria, n	33 (7.1)	59 (21.4)	<0.001
Preoperative ESR, mm/h	8 (4–16)	33 (14–62)	<0.001
Preoperative C-reactive protein, mg/L	0.08 (0.02–0.31)	0.26 (0.09–1.74)	0.010
**Operation and anesthesia details**			
Surgery type, n			
Laparoscopic	27 (5.8)	78 (28.3)	<0.001
Hand-assisted laparoscopic	—	22 (8.0)	
Robot-assisted	72 (15.5)	3 (1.1)	
Open	367 (78.8)	173 (62.7)	
Clinical stage			
T 1/2/3/4/5/6/7/8/9	400/51/10/0/0/0/0/0/2	111/50/62/15/14/11/1/7/3	<0.001
N 0/1	462/2	246/28	
M 0/1	459/5	249/25	
Operation time, hour	2.33 (1.75–3.00)	2.23 (1.67–3.00)	0.450
Anesthesia technique			
Inhalational agent, n	412 (88.4)	263 (95.3)	0.002
Total intravenous agent, n	54 (11.6)	13 (4.7)	0.002
Renal ischemic time, min	24 (17–31)	—	—
Intraoperative mean blood pressure, mmHg	65 (60–71)	66 (61–70)	0.675
Intraoperative dopamine/dobutamine infusion, n	—	1 (0.4)	—
Intraoperative phenylephrine/norepinephrine infusion, n	5 (1.1)	4 (1.4)	0.878
Bleeding and transfusion amount			
pRBC transfusion, n	39 (5.3)	53 (7.1)	<0.001
pRBC transfusion, units	0 (0–0)	0 (0–0)	<0.001
Estimated blood loss, ml	200 (100–350)	200 (100–450)	0.477
Input and output during surgery			
Intraoperative average urine flow rate, ml/kg/hr	1.11 (0.60–1.78)	0.77 (0.37–1.56)	<0.001
Crystalloid administration, ml	1200 (800–1650)	1150 (800–1600)	0.815
Colloid administration, ml	0 (0–300)	0 (0–500)	0.026
Net fluid balance during surgery, ml/kg	12.9 (8.1–18.0)	13.9 (8.6–22.3)	0.022
Outcome			
Length of hospital stay, days	5 (5–6)	6 (5–8)	0.048
Complications			
Urine leakage, n	3 (0.6)	3 (1.1)	0.515
Prolonged ileus, n	7 (1.5)	2 (0.7)	0.350
Wound infection, n	7 (1.5)	3 (1.1)	0.635
Retroperitoneal abscess, n	1 (0.2)	1 (0.4)	0.708
Pneumonia, n	—	1 (0.4)	0.194

The values are expressed as the median [interquartile range] or number (%).

AKI = acute kidney injury; GFR = glomerular filtration rate; pRBC = packed red blood cell; FFP = fresh frozen plasma; ESR = erythrocyte sedimentation rate.

Net fluid balance was calculated by total input subtracted by total output.

After excluding patients with preoperative estimated GFR < 30 ml/min/1.73 m^2^ or anuria or missing creatinine values, a total of 742 patients met the inclusion criteria of this study (partial nephrectomy: 466, radical nephrectomy: 276). The incidence of AKI was 14.4% (67/466) [stage 1: 59 (12.7%); stage 2: 3 (0.6%); stage 3: 5 (1.1%)] after partial nephrectomy and 57.6% (159/276) [stage 1: 149 (54.0%); stage 2: 6 (2.2%); stage 3: 4 (1.4%)] after radical nephrectomy. The incidence of AKI after open partial nephrectomy was 15.0% (55/367) and the incidence after open radical nephrectomy was 53.2% (92/173).

**Figure 1:**
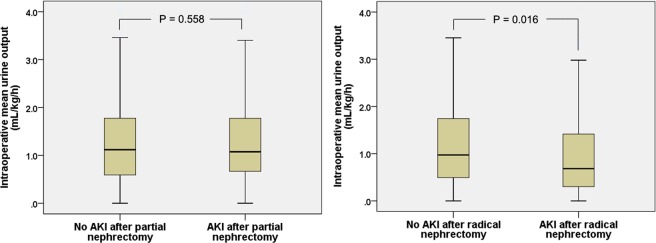
Distribution of intraoperative mean urine output during partial (left) and radical (right) nephrectomy. Each figure compares the distribution of urine output between the patients with and without acute kidney injury (AKI). The thick line and each border in the box show the median, 25^th^ and 75^th^ percentile with the whisker shows the 10^th^ and 90^th^ percentile.

The distribution of intraoperative mean urine output was compared between the patients who underwent partial and radical nephrectomy (Supplemental Fig. S1). The distribution of intraoperative mean urine output was compared between the patients with and without AKI after partial and radical nephrectomy, respectively (Fig. 1). There were significant differences in the distribution of urine output between those with and without AKI after radical nephrectomy (*P* = 0.016), while there was no significant difference after partial nephrectomy (*P* = 0.558).

For partial nephrectomy, multivariable logistic regression analysis showed that renal ischemic time during surgery and intraoperative transfusion were significantly associated with AKI (renal ischemic time per 10 min: multivariable adjusted odds ratio [OR] 1.45, 95% confidence interval [CI] 1.12–1.87, *P* = 0.005) (Table 2). Intraoperative mean urine output during partial nephrectomy was not associated with AKI. Baseline eGFR was not associated with postoperative AKI for partial nephrectomy. Nagelkerke’s R^2^ was 0.21 and the resulting logistic regression model fit our data well (Hosmer-Lemeshow goodness-of-fit, chi-square = 4.87, *P* = 0.85).

**Table 2:** Multivariable logistic regression analysis to predict acute kidney injury after partial nephrectomy (n = 466).

Variable	Odds Ratio	95% CI	p-value
Age	0.98	0.95–1.00	0.090
Female	0.65	0.09–1.25	0.865
Body-mass index, kg/m^2^	1.05	0.95–1.15	0.363
Hypertension, n	1.06	0.52–2.14	0.879
Diabetes mellitus, n	0.58	0.22–1.52	0.267
History of cerebrovascular accident, n	2.26	0.37–16.68	0.374
Preoperative hemoglobin, g/dL	0.94	0.78–1.14	0.527
Preoperative albumin, mg/dL	0.68	0.37–1.25	0.209
Preoperative glomerular filtration rate, mL/min/1.73 m^2^	1.00	0.98–1.01	0.624
Preoperative proteinuria, n	1.05	0.99–1.11	0.098
Total intravenous anesthesia, n	0.62	0.21–1.82	0.382
Open vs. laparoscopic or robotic surgery	3.17	1.30–7.73	0.011
Operation time, per hour	1.65	1.07–2.54	0.022
Crystalloid administration, per 1 L	1.27	0.75–2.15	0.367
Colloid administration, per 100 ml	1.04	0.94–1.14	0.488
Transfusion, n	5.40	1.91–15.25	0.001
Renal ischemic time, per 10 min	1.45	1.12–1.87	0.005
Intraoperative mean blood pressure, mmHg	0.97	0.85–1.24	0.752
Intraoperative vasopressor infusion, n	1.10	0.91–1.33	0.677
Intraoperative mean urine flow rate, ml/kg/h	1.07	0.84–1.36	0.600

CI = confidence interval.

For radical nephrectomy, multivariable logistic regression analysis showed that history of hypertension, baseline eGFR and intraoperative mean urine output were significantly associated with postoperative AKI (intraoperative mean urine output: multivariable adjusted OR 0.85, 95% CI 0.73–0.97, *P* = 0.045) (Table 3). Nagelkerke’s R^2^ was 0.22 and the resulting logistic regression model fit our data well (Hosmer-Lemeshow goodness-of-fit, chi-square = 6.75, *P* = 0.58).

**Table 3:** Multivariable logistic regression analysis to predict acute kidney injury after radial nephrectomy (n = 276).

Variable	Odds Ratio	95% CI	p-value
Age	1.00	0.98–1.03	0.968
Female	0.61	0.17–1.27	0.751
Body-mass index, kg/m^2^	1.07	0.97–1.18	0.154
Hypertension, n	2.13	1.13–4.02	0.019
Diabetes mellitus, n	2.02	0.91–4.51	0.085
History of cerebrovascular accident, n	1.77	0.70–6.70	0.811
Preoperative hemoglobin, g/dL	1.23	0.92–1.59	0.092
Preoperative albumin, mg/dL	0.74	0.39–1.40	0.742
Preoperative glomerular filtration rate, mL/min/1.73 m^2^	1.03	1.01–1.05	0.002
Preoperative proteinuria, n	1.09	1.02–1.16	0.026
Total intravenous anesthesia, n	1.35	0.34–5.33	0.672
Open vs. laparoscopic or robotic surgery	0.66	0.36–1.21	0.182
Operation time, h	0.87	0.67–1.13	0.299
Crystalloid administration, per 1 L	0.93	0.71–1.22	0.602
Colloid administration, ml	1.02	0.94–1.10	0.664
Transfusion, n	1.69	0.64–4.43	0.287
Intraoperative mean blood pressure, mmHg	0.95	0.75–1.30	0.658
Intraoperative vasopressor infusion, n	1.05	0.81–1.47	0.751
Intraoperative mean urine flow rate, ml/kg/h	0.85	0.73–0.97	0.045

CI = confidence interval.

Sensitivity analysis after excluding laparoscopic and robot-assisted cases showed that intraoperative mean urine output was not significantly associated with AKI in partial nephrectomy (n = 367) and was significantly associated with AKI in radical nephrectomy (n = 173) (Supplemental Tables S1 and S2).

Multivariable logistic regression analysis using different cutoffs of oliguria during radical nephrectomy showed that mean urine output <1.0 mL/kg/h was a significant predictor of AKI with minimal *P*-value (OR 1.72, 95% CI 1.22–2.86, *P* = 0.035) (Table 4), although mean urine output <0.5 or <0.3 was also significantly associated with AKI. However, there were no significant cutoffs of oliguria during partial nephrectomy that is associated with postoperative AKI.

**Table 4:** Odds ratios (95% confidence intervals) and their *P*-values according to the categorized intraoperative urine flow rate with different cutoffs determined by both the univariable and multivariable logistic regression analysis for acute kidney injury of all stages after partial and radical nephrectomy.

Cutoff	Unadjusted OR (95% CI)	*P*-value	Adjusted OR (95% CI)	*P*-value
**Partial nephrectomy**				
<3.0 ml/kg/h	0.83 (0.38–1.78)	0.626	0.89 (0.33–2.43)	0.822
<2.5 ml/kg/h	0.90 (0.46–1.77)	0.754	0.75 (0.32–1.73)	0.498
<2.0 ml/kg/h	1.17 (0.61–2.23)	0.644	1.10 (0.50–2.40)	0.819
<1.5 ml/kg/h	0.86 (0.50–1.48)	0.592	0.77 (0.41–1.47)	0.429
<1.0 ml/kg/h	0.92 (0.55–1.56)	0.756	0.86 (0.47–1.60)	0.642
<0.5 ml/kg/h	0.56 (0.27–1.18)	0.127	0.58 (0.24–1.41)	0.577
<0.3 ml/kg/h	0.73 (0.32–1.67)	0.450	0.89 (0.33–2.43)	0.822
**Radical nephrectomy**				
<3.0 ml/kg/h	1.69 (0.73–3.91)	0.223	1.58 (0.57–4.38)	0.377
<2.5 ml/kg/h	1.34 (0.66–2.72)	0.425	1.08 (0.46–2.53)	0.859
<2.0 ml/kg/h	0.98 (0.53–1.83)	0.958	0.79 (0.37–1.66)	0.527
<1.5 ml/kg/h	1.36 (0.79–2.34)	0.272	1.16 (0.60–2.23)	0.663
<1.0 ml/kg/h	1.86 (1.14–3.04)	0.013	1.72 (1.22–2.86)	0.035
<0.5 ml/kg/h	1.74 (1.02–2.96)	0.041	1.88 (1.15–3.69)	0.040
<0.3 ml/kg/h	2.05 (1.08–3.88)	0.028	2.00 (1.10–4.14)	0.044

OR = odds ratio, CI = confidence interval. In multivariable logistic regression analysis, all the covariates used in Table 2 were considered.

**Figure 2:**
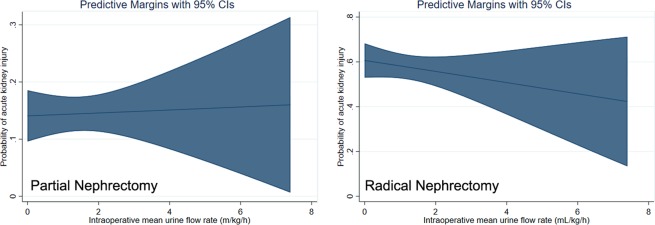
Cubic spline function curves of the multivariable-adjusted relationship between intraoperative mean urine output and the probability of postoperative acute kidney injury after partial (left) and radical (right) nephrectomy. Shaded area represents 95% confidence intervals. Adjusted variables included age, body-mass index, type of surgery, history of hypertension, diabetes mellitus, preoperative hemoglobin, preoperative albumin, preoperative glomerular filtration rate, total intravenous anesthesia with propofol and remifentanil, intraoperative fluid and colloid intake, operation time, and transfusion amount.

Cubic spline function curve showed that intraoperative urine output showed a linear relationship with a negative slope with risk of AKI after radical nephrectomy, while there were no associations between urine output and AKI after partial nephrectomy (Fig. 2).

## Discussion

We evaluated the risk factors to predict postoperative AKI in patients who underwent radical and partial nephrectomy as separate study groups. Since all patients who underwent partial nephrectomy receive intraoperative mannitol infusion and the patient characteristics are completely different between these two surgery groups, we analyzed the radical and partial nephrectomy separately. There was no significant association between intraoperative oliguria and postoperative AKI after partial nephrectomy and there was no cutoff of oliguria that was significantly associated with AKI. However, intraoperative mean urine output was significantly associated with AKI after radical nephrectomy and there were cutoffs including a conventional cutoff of <0.5 and <0.3 mL/kg/h that is significantly associated with the risk of AKI. Oliguria <1.0 mL/kg/h was determined to be optimal cutoff with minimal *P*-value during radical nephrectomy that is associated with elevated risk of AKI.

Several studies have attempted to find an association between oliguria and postoperative AKI^19^ or to derive urine output thresholds that identify AKI after surgery^16–18^. However, these studies were conducted for surgeries that do not include nephrectomy. Using a methodology that was similar to ours, a previous retrospective study in cardiac surgical patients undergoing cardiopulmonary bypass identified a urine flow rate of  1.5 mL/kg/h as a cut-off that was associated with AKI risk^16^. Another retrospective study involving major abdominal surgery reported that <0.3 mL/kg/h was the threshold of the risk of AKI^17^. As such, the threshold urine output to diagnose AKI may vary depending on the different surgical setting and different intraoperative fluid management strategy such as fluid restriction for early recovery after surgery^20^. Our study demonstrated that the association between intraoperative urine output and postoperative AKI could be completely different even among very similar surgeries of partial and radical nephrectomy.

There have been many studies investigating the postoperative renal outcomes after nephrectomy. Rajan *et al*.^1^ investigated the short-term renal outcomes after nephrectomy included the intraoperative urine output as a potential risk factor. However, there was no association between intraoperative urine output and postoperative eGFR or serum creatinine. Other studies investigating short-term or long-term renal outcomes after nephrectomy did not include the urine output as a covariate^2,10,12,14^.

Mannitol is commonly infused intraoperatively during partial nephrectomy to protect renal function from ischemia-reperfusion injury by renal arterial clamping^21^. Mannitol, an osmotic diuretics, influences the renal perfusion and increases the urine output during surgery, thereby influencing the association between mean urine output during surgery and the risk of AKI^22^. Mannitol was suggested to mitigate the effect of ischemic renal injury during partial nephrectomy by increasing renal blood flow. Mannitol may increase renal blood flow through prostaglandin-mediated vasodilatory effect, inducing atrial natriuretic peptide release, or combining both effects^23,24^. However, previous randomized studies reported no significant protective effect of mannitol on the renal function after partial nephrectomy^21,25,26^. Our study results suggest that oliguria threshold of 0.5 or 0.3 mL/kg/h is not significantly associated with the risk of AKI after partial nephrectomy. Also, no cutoffs of oliguria up to 3.0 mL/kg/h was not significant both for univariable or multivariable analysis.

A recent study evaluated the contribution of parenchymal mass reduction and ischemic time on the risk of AKI after partial nephrectomy^2^. The proposed new criteria for diagnosing AKI after partial nephrectomy considering only the ischemic insult by removing the effect of parenchymal mass reduction on the increase in serum creatinine. There was a significant association between the AKI grade determined by the proposed criteria and functional recovery after surgery, while not between the AKI grade determined by the RIFLE (Risk, Injury, Failure, loss of function, and end-stage renal disease) criteria and functional recovery. Although we could not determine AKI according to this new criteria due to lack of data regarding parenchymal mass reduction, further study may evaluate the intraoperative urine output with AKI determined by this new criteria.

Cases with robot-assisted partial nephrectomy were included in our analysis. Robot-assisted partial nephrectomy was reported to have lower morbidity and may achieve similar short-term renal functional outcomes to open surgery^27^. However, in our analysis, there was no significant difference in the incidence of AKI among different surgical types including laparoscopic surgery or robot-assisted surgery. This may be due to the relatively small number of robot-assisted cases, which limits the power to detect the difference in the clinical outcomes. Furthermore, urine output during laparoscopic or robot-assisted nephrectomy is expected to be decreased by the increased intraabdominal pressure during surgery possibly because increased intraabdominal pressure may decrease renal blood flow^28^. This may result in a change in intraoperative urine output and may bias our study results. Although we performed our analysis after excluding laparoscopic or robot-assisted surgery, a small number of cases precludes firm conclusion and further prospective studies with sufficient power are required.

Our study results may be applied to the daily anesthesia practice during partial and radical nephrectomy, although prospective studies are required to validate our results. Oliguria of <1.0 mL/kg/hr during radical nephrectomy may alarm the anesthesiologists who care the patients to increase preload by crystalloid administration or increase cardiac output. However, oliguria during partial nephrectomy with mannitol infusion may not be relevant and other monitoring for AKI may be required such as urine or serum biomarker^29,30^. Although oliguria during surgery was associated with increased risk of AKI after radical nephrectomy, it is not certain whether increasing urine output by increasing preload or cardiac output may decrease postoperative AKI. As AKI is considered to develop by decreased renal perfusion and ischemia-reperfusion injury^31^, further studies are required to evaluate the hypothesis that any measures for hemodynamic optimization may decrease the risk of AKI.

Other potentially modifiable risk factors of our study results include the renal ischemic time and transfusion for partial nephrectomy^32^. Time and type of ischemia are considered to be crucial factors associated with partial nephrectomy^32,33^. The risk factors of renal ischemic time and duration of surgery were consistent with a previous study which found their significant association with postoperative renal function measured by eGFR^2^. Intraoperative transfusion was a significant risk factor of AKI After partial nephrectomy in our study, while previous studies did not report this association^1,2^. This may be due to the different incidence of transfusion and transfusion amount or study power. Intraoperative transfusion is known to be a significant risk factor of AKI after cardiac surgery^30,34^.

For radical nephrectomy, the preoperative baseline renal function measured by eGFR was significantly associated with postoperative AKI, while not for partial nephrectomy in our study. This may be because there were more patients with poor baseline eGFR for radical nephrectomy, which result in increased discriminative power to differentiate the low and high risk of AKI, while not for the patients with partial nephrectomy.

The present study had several limitations. Firstly, it was a single-center retrospective analysis. The measurement of urine flow rate was based on our medical records, which may be imprecise. Furthermore, our cut-off for urine output may not be extrapolated to other institutions with different fluid management strategy and different baseline medical conditions, although multivariable adjustment of fluid and colloid administration during surgery was performed in this study. The intraoperative urine output may differ markedly depending on the intraoperative goal of fluid management and transfusion. Secondly, in our analysis, we used a mean urine flow rate during surgery rather than hourly urine output. However, there may be critical periods during nephrectomy, such as renal arterial clamping period, during which the urine flow rate may be closely related to the risk of AKI. Duration of oliguria may also impact the risk of AKI; in future studies, this could be evaluated in patients who experienced different oliguria durations and in whom different oliguria cut-offs were used, including 1.0 mL/kg/h. Thirdly, different techniques of surgery including open, laparoscopic, and robotic surgeries were included in our analysis. Although we investigated the type of surgery and adjusted this factor in our multivariable analysis, this may act as a potential confounding factor. Fourthly, the baseline renal function was significantly different between the patients with partial and radical nephrectomy. Although we performed multivariable analysis, this may affect the incidence of postoperative AKI.

In conclusion, we analyzed the risk factor for postoperative AKI after partial and radical nephrectomy. Intraoperative mean urine output <1.0 mL/kg/h during radical nephrectomy was identified as the optimal oliguria threshold associated with postoperative AKI. However, during partial nephrectomy during which mannitol is infused, intraoperative mean urine output was not associated with AKI at any cutoff investigated. Intraoperative oliguria may have different clinical implication regarding AKI after partial and radical nephrectomy.

## Methods

This retrospective observational study was approved by the institutional review board of Seoul National University Hospital (1803- 041- 926). Written informed consent was waived due to the retrospective nature of the present study. All methods were carried out in accordance with the approved guidelines and regulations. We reviewed electronic medical records of 1108 patients who were ≥18 years old, had a renal mass and underwent radical (n = 276, 37.2%) or partial (n = 466, 62.8%) nephrectomy regardless of surgical techniques at Seoul National University Hospital between May 1, 2010 and June 30, 2014. The patients with preoperative estimated GFR < 30 ml/min/1.73 m^2^ or anuria or missing baseline creatinine values were excluded. The patients with poor baseline renal function were excluded to evaluate the effect of oliguria on the risk of AKI.

The anesthesia protocol of our institution during the study period was as follows. Anesthesia was induced with propofol, rocuronium, and remifentanil or fentanyl. Anesthesia was maintained either by sevoflurane or total intravenous anesthesia with propofol and remifentanil. Volume controlled ventilation was maintained with a tidal volume of 6–8 ml/kg and a FiO2 of 0.4 to 0.5. All patients who underwent partial nephrectomy received intraoperative mannitol infusion (20 g) within 30 min prior to renal vascular clamping. No patients who underwent radical nephrectomy received intraoperative mannitol during surgery.

Demographic, baseline medical history, laboratory data, anesthesia and surgery-related parameters that were known to be associated with AKI after nephrectomy were extracted from our electronic medical record (Table 1)^1,2,35^. According to the surgical technique, 540 patients (72.8%) underwent open nephrectomy, 105 patients (14.2%) underwent laparoscopic surgery, 75 patients (10.1%) robot-assisted surgery, and 22 patients (3.0%) underwent hand-assisted laparoscopic surgery.

The primary outcome of our study was AKI after any type of nephrectomy. Postoperative AKI was defined by the creatinine criteria of Kidney Disease: Improving Global Outcomes (KDIGO) criteria, which was determined according to the maximal change in serum creatinine level during the first seven postoperative days^15,30^. The most recent serum creatinine level measured before surgery was used as the baseline value.

### Statistical analysis

SPSS software version 23.0 (IBM Corp., Armonk, NY, USA) and STATA/MP version 15.1 (StataCorp, College Station, TX, USA) were used to analyze the data. For all analyses, *P* < 0.05 was considered statistically significant. The Shapiro-Wilk test was used to determine the normality of the data. The following three analyses were performed separately for partial and radical nephrectomy.

First, the distribution of intraoperative urine output during surgery was investigated according to the different surgical type and development of AKI. Second, we examined the relationship between intraoperative urine output and the risk of AKI through multivariable logistic regression analysis after radical or partial nephrectomy, separately. The following variables of patient demographics and surgery-related variables were used to adjust for the association between intraoperative mean urine output and AKI: age, gender, body-mass index, type of surgery, history of hypertension, diabetes mellitus, preoperative hemoglobin, preoperative albumin, preoperative glomerular filtration rate, total intravenous anesthesia with propofol and remifentanil, intraoperative fluid and colloid intake, intraoperative mean blood pressure, intraoperative vasopressor use, operation time and intraoperative transfusion amount. The intraoperative mean urine output was used as a continuous variable in this analysis. Neither stepwise variable selection nor univariable screening was performed for logistic regression analysis. The performance of logistic regression model was evaluated by Nagelkerke’s R^2^ and model fit was evaluated by Hosmer-Lemeshow goodness-of-fit statistics. To address potential confounding by type of surgery, we performed the logistic regression analysis after excluding all cases with laparoscopic or robot-assisted surgery as a sensitivity analysis.

Third, multivariable logistic regression analysis was performed again using different cutoffs of intraoperative mean urine output to find the optimal threshold of oliguria with minimal p-value approach. This analysis was also performed separately for partial and radical nephrectomy. Same covariates were used to adjust for potential confounding factors. Fourth, a cubic spline function was drawn to identify any linear or non-linear relationship between intraoperative mean urine output and the risk of AKI and to identify the inflection point where the risk of AKI increase. Missing values were present in less than 1% during our data collection.

Sample size was not calculated preliminary, but was validated by post-calculation to detect independent predictors in logistic regression analysis for partial and radical nephrectomy, respectively. We used the observed incidence of AKI in our study patients of 14.4% and 57.6% for partial and radical nephrectomy, respectively, and the number of significant covariates of five. Based on the rule of 10 patients with an outcome of interest per each predictor^36^, 347 patients and 87 patients or more were required for each dataset of partial and radical nephrectomy.

## Supplementary information

Supplementary materials
