# Supplementary material for: The association between intraoperative urine output and postoperative acute kidney injury differs between partial and radical nephrectomy
Source: Sci Rep. 2019 Jan 24;9:760. doi: 10.1038/s41598-018-37432-7 (PMC6345871; doi:10.1038/s41598-018-37432-7)
Supplement: Supplementary file 1 — Supplementary materials [file 41598_2018_37432_MOESM1_ESM.pdf]

The association between intraoperative urine output and postoperative acute kidney injury differs between partial and radical nephrectomy

: Supplemental materials

Min Hur[1], **Sun-Kyung Park**[1], **Seokha Yoo**[1], **Sheung-Nyoung Choi**[1], **Chang Wook Jeong**[2], **Won Ho Kim**[1], **Jin-Tae Kim**[1], **Cheol Kwak** [2], **Jae-Hyon Bahk**[1]

[1]Department of Anesthesiology and Pain Medicine, Seoul National University Hospital, Seoul National University College of Medicine

[2]Department of Urology, Seoul National University Hospital, Seoul National University College of Medicine

	Title	Page
**Supplemental Figure S1.**	Histogram showing the distribution of intraoperative mean urine output during partial (left upper) and radical (right upper) nephrectomy. Box and whisker plot (lower) shows the same data.	2
**Supplemental Figure S2.**	Histogram showing the distribution of intraoperative mean urine output during laparoscopic/robot-assisted (left upper) and open (right upper) nephrectomy. Box and whisker plot (lower) shows the same data.	3
**Supplemental Table S1.**	Multivariable logistic regression analysis to predict acute kidney injury after open partial nephrectomy (n = 367)	4
**Supplemental Table S2.**	Multivariable logistic regression analysis to predict acute kidney injury after open radial nephrectomy (n = 173)	5

Supplemental Figure S1. Histogram showing the distribution of intraoperative mean urine output during partial (left upper) and radical (right upper) nephrectomy. Box and whisker plot (lower) shows the same data. The thick line and each border in the box show the median, 25th and 75th percentile with the whisker showing the 10th and 90th percentile.

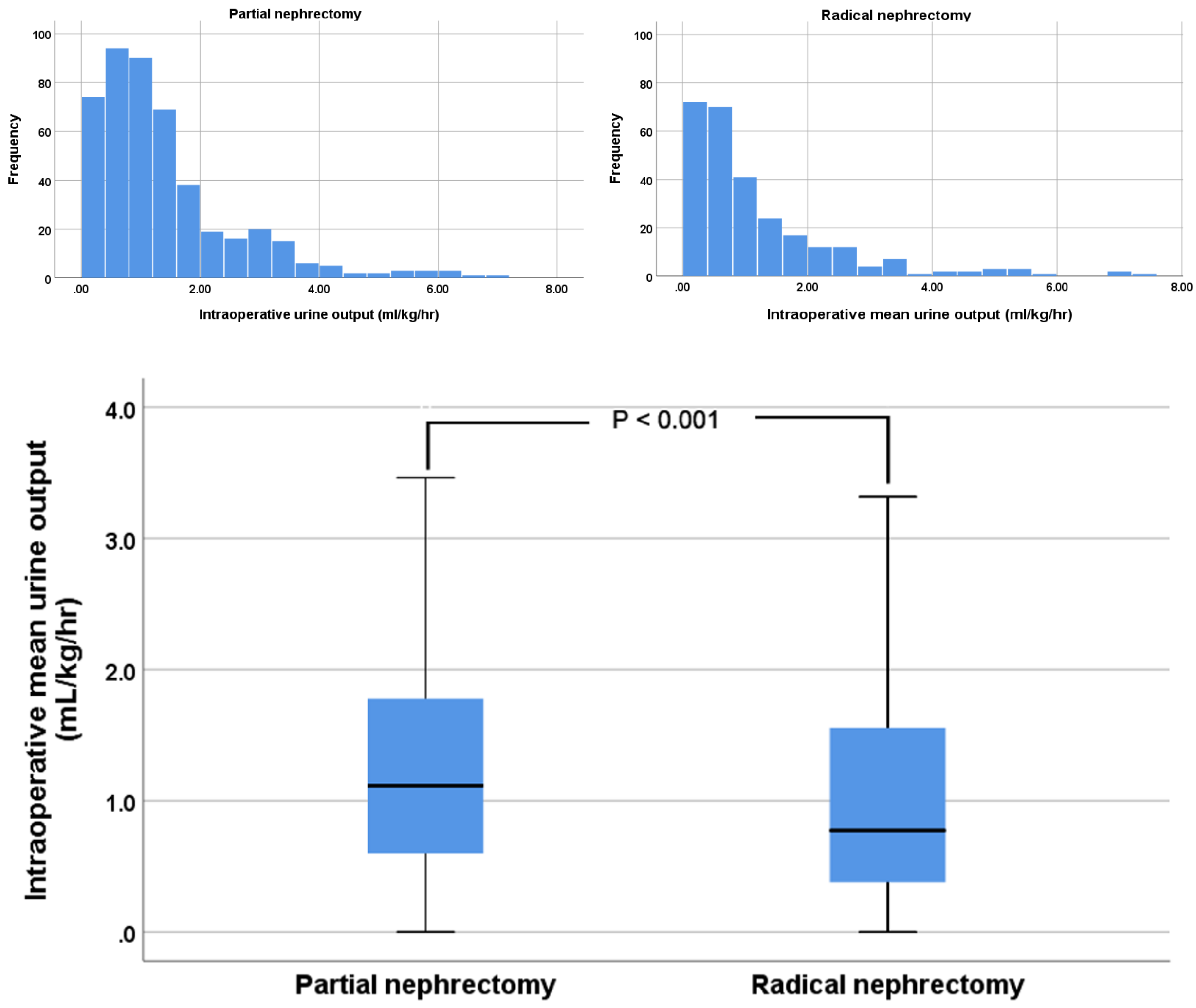

Supplemental Figure S2. Histogram showing the distribution of intraoperative mean urine output during laparoscopic/robot-assisted (left upper) and open (right upper) nephrectomy. Box and whisker plot (lower) shows the same data. The thick line and each border in the box show the median, 25th and 75th percentile with the whisker showing the 10th and 90th percentile.

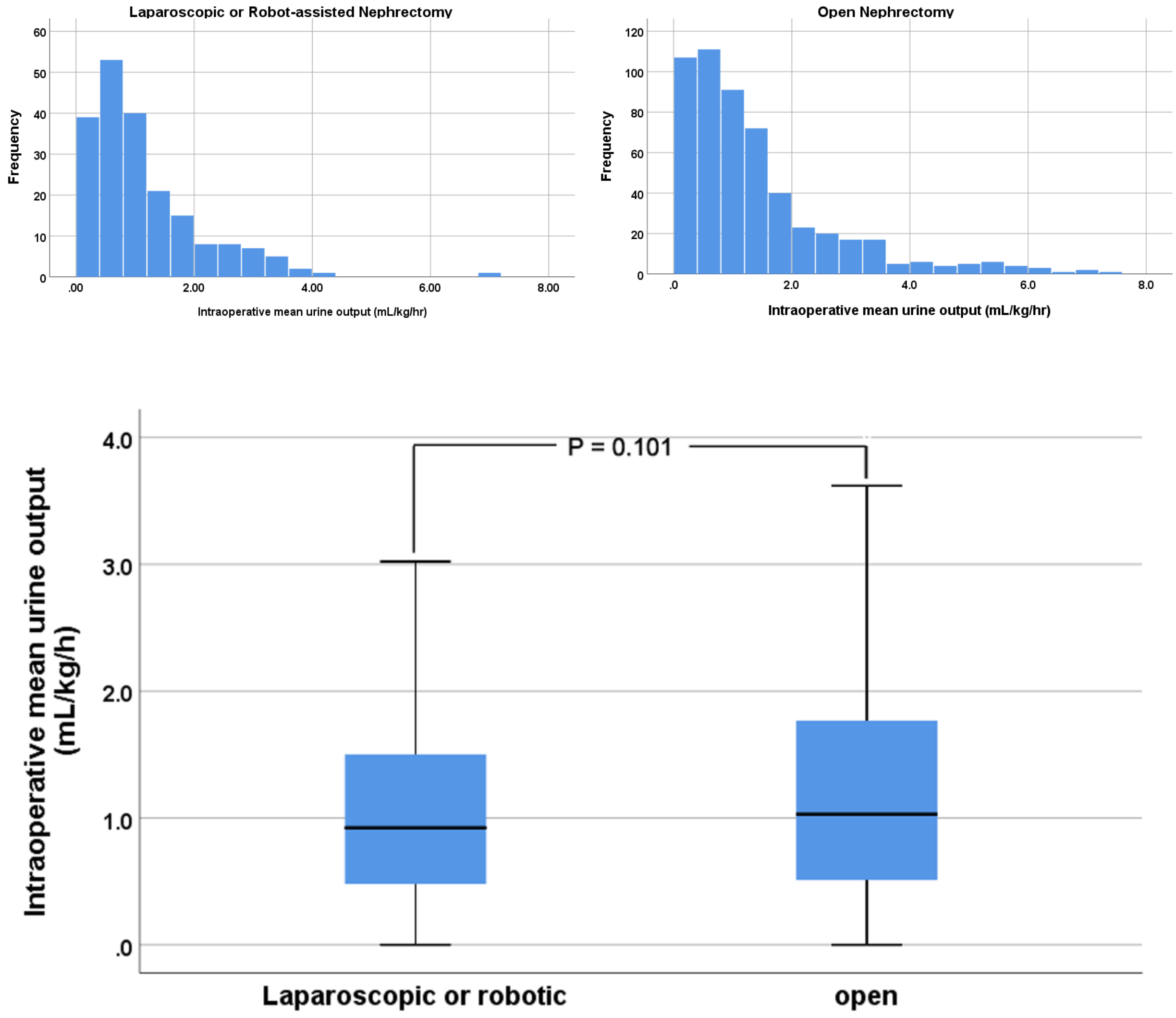

Supplemental Table S1. Multivariable logistic regression analysis to predict acute kidney injury after open partial nephrectomy (n = 367)

Variable	Odds Ratio	95% CI	p-value
Age	0.98	0.95 – 1.01	0.147
Female	0.65	0.12 – 1.16	0.886
Body-mass index, kg/m^2	1.06	0.95 – 1.18	0.296
Hypertension, n	1.11	0.50 – 2.45	0.795
Diabetes mellitus, n	0.58	0.20– 1.65	0.303
History of cerebrovascular accident, n	1.04	0.10 – 11.4	0.973
Preoperative hemoglobin, g/dL	1.02	0.82 – 1.26	0.879
Preoperative albumin, mg/dL	0.59	0.31 – 1.12	0.103
Preoperative glomerular filtration rate, mL/min/1.73 m^2	0.99	0.97 – 1.01	0.431
Preoperative proteinuria, n	1.03	0.96 – 1.10	0.125
Total intravenous anesthesia, n	0.51	0.11 – 2.31	0.384
Operation time, per hour	1.60	1.01 – 2.57	0.048
Crystalloid administration, per 1 L	1.40	0.77 – 2.54	0.266
Colloid administration, per 100 ml	1.00	0.89 – 1.13	0.999
Transfusion, n	6.47	2.08 – 20.16	0.001
Renal ischemic time, per 10 min	1.62	1.21 – 2.17	0.001
Intraoperative mean blood pressure, mmHg	0.95	0.80 – 1.29	0.614
Intraoperative vasopressor infusion, n	1.05	0.72 – 1.54	0.495
Intraoperative mean urine flow rate, ml/kg/h	0.99	0.76 – 1.30	0.960

CI = confidence interval.

Supplemental Table S2. Multivariable logistic regression analysis to predict acute kidney injury after open radial nephrectomy (n = 173)

Variable	Odds Ratio	95% CI	p-value
Age	1.00	0.94 – 1.03	0.943
Female	0.55	0.18 – 1.21	0.639
Body-mass index, kg/m^2	1.07	0.94 – 1.21	0.296
Hypertension, n	3.14	1.43 – 6.91	0.004
Diabetes mellitus, n	2.62	1.02 – 6.73	0.046
History of cerebrovascular accident, n	1.56	0.57 – 5.50	0.619
Preoperative hemoglobin, g/dL	1.08	0.84 – 1.39	0.534
Preoperative albumin, mg/dL	0.90	0.44 – 1.68	0.293
Preoperative glomerular filtration rate, mL/min/1.73 m^2	1.03	1.00– 1.05	0.025
Preoperative proteinuria	1.07	1.00 – 1.14	0.041
Total intravenous anesthesia, n	1.60	0.29 – 8.80	0.588
Operation time, h	0.79	0.58 – 1.07	0.124
Crystalloid administration, per 1 L	0.97	0.73 – 1.30	0.858
Colloid administration, ml	1.03	0.95 – 1.12	0.504
Transfusion, n	1.32	0.46 – 3.80	0.603
Intraoperative mean blood pressure, mmHg	0.89	0.63 – 1.51	0.851
Intraoperative vasopressor infusion, n	1.12	0.51- 1.67	0.812
Intraoperative mean urine flow rate, ml/kg/h	0.80	0.51 – 0.95	0.047

CI = confidence interval.
